# Assessment of Chronic Myeloid Leukaemia In Vitro Models Variability: Insights Into Extracellular Vesicles

**DOI:** 10.1111/jcmm.70901

**Published:** 2025-10-22

**Authors:** Silvia Mutti, Alessia Cavalleri, Stefania Federici, Valentina Mangolini, Lucia Paolini, Cristian Bonvicini, Rosalba Monica Ferraro, Elena Laura Mazzoldi, Luca Garuffo, Besjana Xhahysa, Alessandro Leoni, Federica Trenta, Federica Re, Silvia Clara Giliani, Daniele Avenoso, Mirko Farina, Michele Malagola, Domenico Russo, Simona Bernardi

**Affiliations:** ^1^ Department of Clinical and Experimental Sciences University of Brescia, Unit of Blood Diseases and Bone Marrow Transplant, ASST Spedali Civili Brescia Italy; ^2^ Centro di Ricerca Emato‐Oncologica AIL (CREA) ASST Spedali Civili di Brescia Brescia Italy; ^3^ Department of Mechanical and Industrial Engineering University of Brescia Brescia Italy; ^4^ National Interuniversity Consortium of Materials Science and Technology (INSTM) Florence Italy; ^5^ Department of Molecular and Translational Medicine (DMMT) Università di Brescia Brescia Italy; ^6^ IRCCS Fondazione Don Carlo Gnocchi ONLUS Milan Italy; ^7^ Department of Medical and Surgical Specialties, Radiological Sciences and Public Health (DSMC) Università di Brescia Brescia Italy; ^8^ Center for Colloid and Surface Science (CSGI) Sesto Fiorentino Italy; ^9^ Molecular Markers Laboratory IRCCS Istituto Centro San Giovanni di Dio Fatebenefratelli Brescia Italy; ^10^ Department of Molecular and Translational Medicine University of Brescia, “Angelo Nocivelli” Institute for Molecular Medicine, ASST Spedali Civili Brescia Italy; ^11^ National Center for Gene Therapy and Drugs Based on RNA Technology (CN3) Padua Italy

**Keywords:** chronic myeloid leukaemia, extracellular vesicles, Fourier‐transform infrared spectroscopy, K562, KCL22, tyrosine kinase inhibitors

## Abstract

Chronic Myeloid Leukaemia is driven by the BCR::ABL1 fusion gene. Although Tyrosine Kinase Inhibitors have significantly improved patient outcomes, drug resistance and disease persistence remain challenges, highlighting the need for effective preclinical models. We observed cellular heterogeneity among CML models in response to TKIs, influencing viability, metabolism, and molecular markers. With growing interest in extracellular vesicles as mediators of leukaemia progression via oncogenic cargo like BCR::ABL1, we explored whether EVs from different CML cell lines exhibit distinct features. EVs from K562 and KCL22 cells were characterised under basal conditions using Fourier Transform Infrared spectroscopy, Atomic Force Microscopy, dot blotting, and Nanoparticle Tracking Analysis. We assessed EV release and BCR::ABL1 content before and after treatment with imatinib, nilotinib, or dasatinib, alongside Ki67 expression to gauge proliferation. Fourier Transform Infrared Spectroscopy with Principal Component Analysis revealed distinct clustering of EVs by cell line. While untreated K562 and KCL22 cells showed similar EV output and BCR::ABL1 content, post‐treatment K562 cells released more EVs with elevated BCR::ABL1 transcripts. KCL22 cells showed earlier reduction in Ki67 expression. These findings highlight model‐dependent EV behaviour, reflecting patient heterogeneity and reinforcing the need for careful model selection in CML research.

## 1 Introduction

Chronic Myeloid Leukaemia (CML) is a myeloproliferative disorder driven by the *BCR::ABL1* fusion gene, which arises from the Philadelphia chromosome (Ph+). This fusion gene encodes a constitutively active tyrosine kinase that promotes uncontrolled proliferation and prolonged survival of myeloid cells [1, 2, 3]. The disease is naturally characterised by a multistep course beginning with a chronic phase, which, in the absence of treatment, progresses to a terminal blastic phase. Traditionally, an intermediate accelerated phase was also described, though it has been recently abrogated in some guidelines [4]. The advent of targeted therapies with tyrosine kinase inhibitors (TKIs) has revolutionised CML management, substantially improving disease control and altering its natural course in the majority of patients. Imatinib, the first‐generation TKI, along with second‐ and third‐generation inhibitors such as nilotinib, dasatinib, bosutinib, and ponatinib, has markedly improved patient outcomes by effectively suppressing the oncogenic activity of BCR::ABL1 [5, 6]. However, significant clinical challenges persist, including therapy resistance and toxicity, disease persistence, and the need for more refined preclinical models to better predict therapeutic responses [7, 8, 9, 10].

Recent research from our group underscored the pronounced heterogeneity in cellular responses among widely used CML cell lines (K562, LAMA84, and KCL22) treated with clinically approved TKIs (imatinib, nilotinib, dasatinib, bosutinib, ponatinib, and asciminib) [11]. Employing a multiparametric workflow, we identified key differences in cellular behaviour, highlighting the importance of accounting for cellular heterogeneity in CML research to improve the translational relevance of preclinical models [12].

Extracellular vesicles (EVs) have now widely emerged as pivotal mediators of intercellular communication within the tumour microenvironment and have been recently explored in some haematological malignancies [13, 14, 15, 16, 17]. In CML, EVs derived from leukaemic cells influence various aspects of disease biology, including modulation of immune response, promotion of angiogenesis, and facilitation of metastasis [18]. These vesicles carry bioactive molecules, such as proteins, lipids, and RNA, which can profoundly impact recipient cells [19, 20]. Notably, CML‐derived EVs have been shown to transfer the *BCR::ABL1* fusion gene to other cells, potentially driving disease progression and therapy resistance [21, 22, 23]. K562‐derived microvesicles, enriched in transcripts related to leukaemic functions and extracellular communication, have been shown to transfer oncogenic signals to healthy mesenchymal stem cells, enhancing their proliferation [24].

While these findings underscore the functional importance of EVs in CML pathogenesis, they also highlight the technical challenges associated with their study. EVs are a highly heterogeneous population that varies in size, composition, and biogenesis, making their standardisation, isolation, and characterisation challenging across experimental models. In this context, advanced analytical approaches are required to resolve their complexity and gain deeper molecular insights. One such approach is Fourier‐transform infrared (FTIR) spectroscopy, which offers a valuable, label‐free, and high‐resolution method for EV analysis [25]. This technique enables the detection of subtle molecular differences that would otherwise remain undetectable with conventional tools. By capturing detailed spectral fingerprints of EV components, FTIR holds promise not only for the classification of vesicle subtypes but also for the identification of disease‐specific signatures and treatment‐induced changes [26, 27, 28, 29]. Thus, integrating such high‐resolution techniques into EV research may significantly enhance our understanding of their biological roles and improve the reliability of EV‐based biomarkers in CML.

Despite the recognised significance of EVs in CML pathophysiology, few studies have examined the vesicular release profiles of drug‐sensitive CML cell lines under TKI treatment. Understanding how treatments influence EVs' release and content could provide crucial insights into their therapeutic mechanisms and the development of resistance. This is particularly important as EVs are becoming more widely acknowledged as novel biomarkers for disease monitoring in patients [30, 31, 32, 33].

In support of our previous study, which highlighted the importance of selecting appropriate in vitro models for preclinical research, this study aims to evaluate the vesicular differences across the two most used CML cell lines K562 and KCL22, both derived from pleural effusions of patients in blast crises. We examined the effects of imatinib, nilotinib, and dasatinib on EVs release and cargo in these cell lines, evaluating different parameters. The selected TKIs are the first three treatments approved for first‐line clinical management of CML and are the most administered in Europe [34, 35, 36, 37, 38]. Specifically, EVs release was quantified, and the vesicular cargo was examined for the presence of *BCR::ABL1*. Additional analyses focused on the evaluation of the cellular proliferation marker *Ki67*. To validate the results, K562 and KCL22‐derived EVs were characterised according to the current MISEV2023 guidelines to ensure rigorous and standardised assessment of their properties [20], via Nanoparticles Tracking Analysis (NTA) in terms of concentration and size distribution, via Atomic Force Microscopy (AFM) to determine the morphological integrity of nanoparticles in the preparations, and dot blot for the presence of vesicular markers. Finally, to account for intrinsic differences in the molecular fingerprint between EVs derived from the two CML models, K562 and KCL22‐derived EVs were characterised via Fourier‐transform infrared (FTIR) spectroscopy.

## 2 Materials and Methods

### 2.1 Characterisation and Quantification of K562 and KCL22‐Derived EVs in Basal Condition

K562‐s (ACC10) and KCL22‐s (ACC519) cell lines were purchased from DSMZ, German Collection of Microorganisms and Cell Cultures. Prior to experimentation, the presence of Mycoplasma in cell cultures was excluded by Polymerase Chain Reaction (PCR) assays.

K562 and KCL22 cell lines were expanded in T75 cm^2^ flasks in complete medium composed of RPMI‐1640 medium (R2405, Sigma‐Aldrich) supplemented with 10% FBS (Gibco, Life Technologies) to obtain at least 2 × 10^8^ cells per line. Then, the medium was replaced with fresh serum‐free medium, and cells were cultured overnight to allow for clean EVs release and harvesting. EVs were isolated via a commercial kit (10 mL of conditioned medium for each cell line) (Cell Culture Media Exosome Purification Kit, #60400, Norgen Biotek Corporation) following the manufacturer's protocol, using the following reagents provided by the kit: ExoC Buffer, Slurry E, and ExoR Buffer. To concentrate EVs, samples were centrifuged at 100,000 g for 2 h at 4°C. Then, EVs were resuspended in 50 μL of double‐distilled water (ddH_2_O). Total protein concentration of the samples was evaluated by Bicinchoninic acid (BCA) Assay Kit (ThermoFisher) following the manufacturer's instructions.

For dot blot analyses, 6 or 3 μg of EVs were spotted on a nitrocellulose membrane and analyzed for the presence of CD63, TSG101, and FLOT‐1 markers as described in Supporting Information [39, 40].

For AFM imaging, 3 μL of EVs samples were spotted onto freshly cleaved mica sheets (PELCO Mica discs Grade V‐1, thickness 0.15 mm, 10 mm diameter from Ted Pella Inc) and dried at RT. Samples were analysed using a Nanosurf NaioAFM equipped with Multi75AI‐G probes. Images were acquired in dynamic mode, scan size ranged from 25 to 2.5 μm and scan speed was set to 1 s/line. AFM images were processed using Gwyddion (64bit).

To evaluate the size distribution and concentration of EVs derived from K562 and KCL22 cell cultures, Nanoparticle Tracking Analysis (NTA) was performed using a NanoSight NS300 instrument equipped with a 488 nm laser (Malvern Panalytical Ltd). Prior to analysis, samples were diluted in freshly filtered phosphate‐buffered saline (PBS) to achieve an optimal particle count per frame (20–120 particles/frame) and introduced into the sample chamber via a syringe pump, ensuring a continuous flow of 50 μL/min. For each sample, five 60‐s videos were recorded and analyzed using NTA software version 3.4 Build 3.4.4.

### 2.2 Fourier‐Transform Infrared (FTIR) Spectroscopy of K562‐ and KCL22‐Derived EVs in Basal Condition

K562‐s and KCL22‐s cells were expanded in complete medium as described in Section 2.1. Upon confluence, cells were collected by centrifugation and sub‐cultured in serum‐free medium for 24 h to promote extracellular vesicle release. For each cell line, four biological replicates consisting of 5 × 10^7^ cells each were prepared. After 24 h, the conditioned medium was collected and EVs were isolated using a commercial kit as previously described. To further concentrate the EVs, samples were subjected to ultracentrifugation at 100,000× *g* for 2 h at 4°C, and the resulting pellets were resuspended in 60 μL of ddH_2_O. FTIR measurements were performed with a Cary 630 spectrometer (Agilent) operating in transmission mode. An Attenuated Total Reflectance (ATR) diamond accessory was used, enabling faster data collection, lower noise, and superior quality. For spectra acquisition, 6 μL of each EVs suspension was deposited onto the ATR diamond window and dried under a stream of nitrogen gas. Spectra were recorded in triplicate from each of the four biological replicates per cell line, in the range of 4000–400 cm^−1^, with a resolution of 4 cm^−1^ and 32 co‐added scans per measurement. Standard Normal Variate normalisation corrected baseline shifts and signal intensity variations, and Principal Component Analysis (PCA) was applied to the full spectral range for dimensionality reduction, using the multivariate data analysis software CAT (R. Leardi, C. Melzi, G. Polotti, CAT (Chemometric Agile Tool), freely downloadable from http://gruppochemiometria.it/ index.php/software).

### 2.3 Cell Culture Conditions Under TKIs Treatment

K562 and KCL22 cell lines were grown in complete medium as previously described [11]. Following expansion in T75 cm^2^ flasks, cells were seeded in a 24‐well plate at a density of 5 × 10^4^ cells/well in 500 μL of complete medium and cultured for 24 and/or 48 h.

Three TKIs from the Adult Marrow Transplant Centre of the ASST Spedali Civili of Brescia were tested at their corresponding concentrations: imatinib (Gleevec, Novartis) 0.64 μM; nilotinib (Tasigna, Novartis) 5 μM; dasatinib (Sprycel1, Bristol‐Myers Squibb) 1.3 nM. Drug concentrations, which aimed to simulate the in vitro effects of the dose administered in clinical practice, were identified in the literature and adapted according to our experience [41, 42, 43].

**FIGURE 1:**
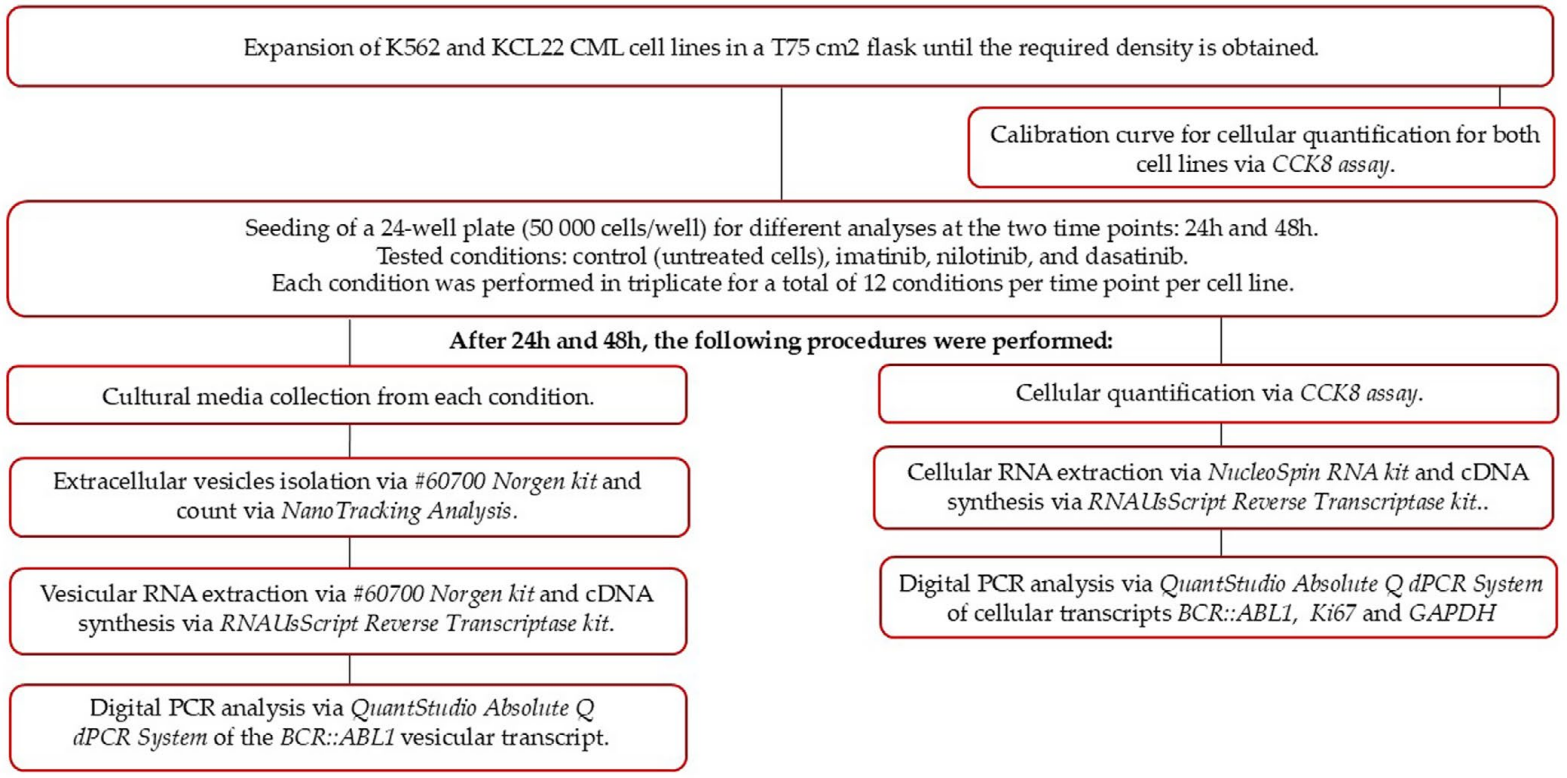
Flowchart representing the experimental design of the study.

Each condition was repeated in triplicate, for a total of 12 conditions for each cell line and each time point. The in vitro experimental investigation scheme is shown in the flow chart below (Figure 1).

### 2.4 Cellular Metabolic Activity Assay, Cellular RNA Extraction and cDNA Synthesis After TKIs Treatment

Cell number following treatments was assessed using the commercial enzyme‐based Cell Counting Kit‐8 (Sigma‐Aldrich) for normalisation. For the procedure, the manufacturer's instructions were followed. Following a two‐hour incubation, absorbance was measured at 460 nm using a Tecan Infinite 200 spectrophotometer (Männedorf). An appropriate calibration curve was used to precisely estimate the number of cells. Subsequently, total cellular RNA from each condition was extracted and reverse‐transcribed to cDNA using the NucleoSpin RNA extraction Kit (Machery‐Nagel) and the RNAUsScript Reverse Transcriptase Kit (LeGene Biosciences) respectively, as previously described [11].

### 2.5 EVs Separation, Quantification and RNA Extraction After TKIs Treatment

Intact EVs and vesicular total RNA isolation were performed via the commercial kit Cell culture media exosome purification and RNA isolation mini kit (500 μL of conditioned medium for each cell line) (#60700, Norgen Biotek Corporation). To ensure the required volume for kit use, 500 μL of fresh complete medium was added to the medium collected from each culture condition. The first centrifugation was carried out at 200× *g* for 15 min to facilitate the removal of any cells and debris. Subsequently, EVs were isolated following the manufacturer's protocol. An aliquot of pure EVs from each condition was stored for extracellular vesicles quantification via NTA. Total vesicular RNA was eluted in 50 μL. Finally, cDNA was synthesised via RNAUsScript Reverse Transcriptase kit (LeGene Biosciences) according to the manufacturer's instructions. The final reaction volume was 20 μL.

EVs were quantified via Nano‐Sight NS300 Instrument (Malvern). Samples were diluted with 0.2 μm filtered 1× PBS to obtain an optimal range of 20–150 particles/frame. For each sample, 5 videos of 60‐s duration were recorded, and data were processed using NanoSight NTA Software 3.2. Post‐acquisition settings were kept constant between samples. Raw concentration data (particles/mL) obtained from NTA were normalised to obtain EVs concentration in the media. The negative control (filtered PBS) included did not interfere with the sample analysis because it was below the detection limit.

### 2.6 dPCR Analysis of Vesicular *BCR::ABL1* and Cellular *Ki67* and *GAPDH* After TKIs Treatment

Vesicular *BCR::ABL1* (Hs03024541_ft, FAM‐MGB) and cellular *Ki67* (Hs04260396_g1, CY5‐MGB) and *GAPDH* (Hs02786624_g1, VIC‐MGB) transcripts were quantified using dPCR on the QuantStudio Absolute Q Digital PCR System (Life Technologies) via multiplexing strategy, as described in Supporting Information. Results were normalised to either *GAPDH* or the number of total cells to correct for pre‐analytical variability in extraction and reverse transcription among triplicates.

### 2.7 Statistical Analysis

The statistical analysis has been performed using the GraphPad Prism software (version 10.0.2). More precisely, the one‐way ANOVA with Sidak's multiple comparisons test was performed to compare the data obtained between the two in vitro cell models tested. Student's t‐test (equal variance not assumed) was performed to determine differences in EV size diameter. Statistical significance was accepted at a probability level of *p* < 0.05.

## 3 Results

### 3.1 Separation and Characterisation of Basal K562 and KCL22‐Derived EVs in Basal Condition

**FIGURE 2:**
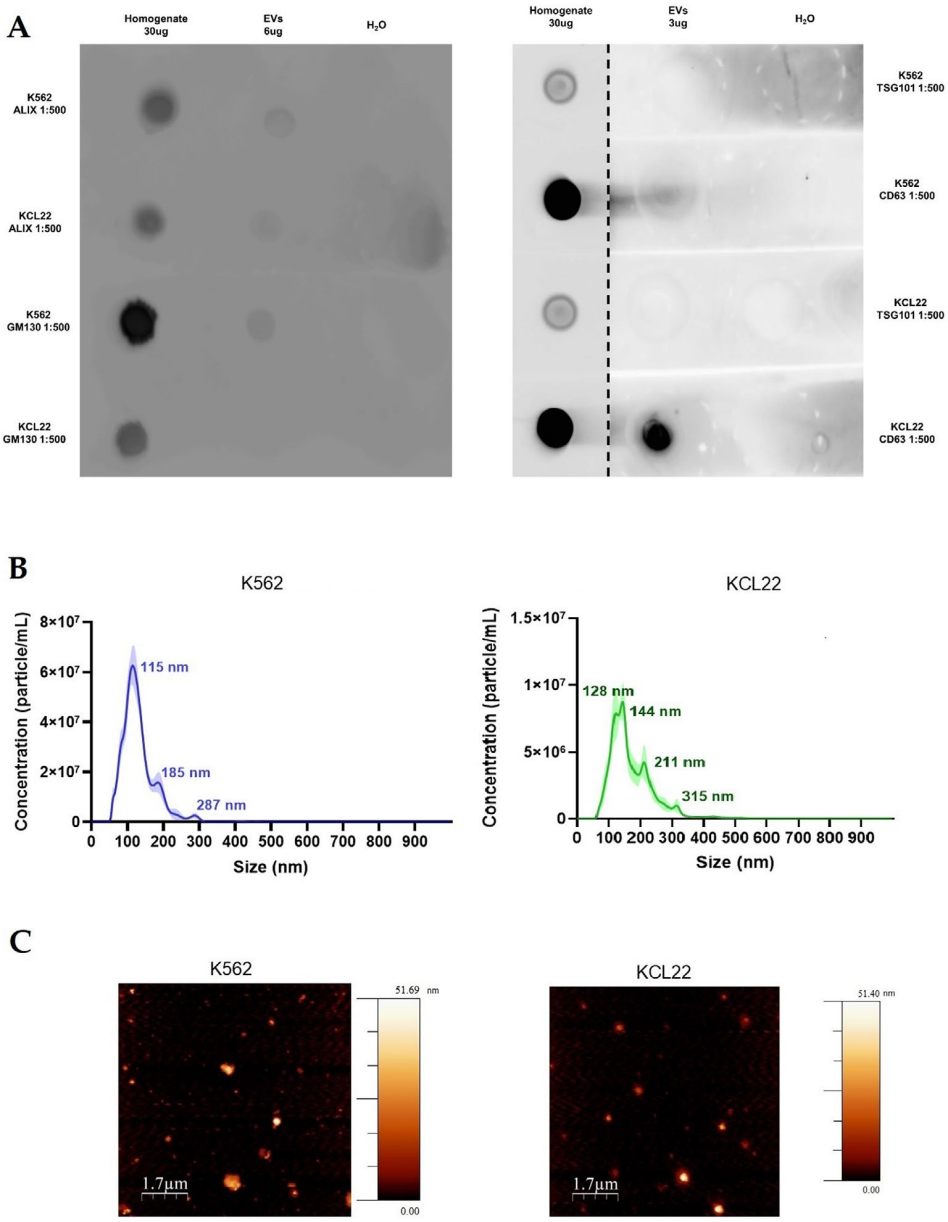
Characterisation of EVs isolated from KCL22 and K562 cell lines. (A) Biochemical characterisation by dot blot. EV samples (6 μg or 3 μg) and total cell homogenate (30 μg) from KCL22 and K562 cell lines were spotted onto nitrocellulose membrane and analysed for the indicated antibodies. ddH20 (H20, 10 μL) was used as negative control. (B) Size distribution profiles of EVs from KCL22 and K562 cells by NTA. (C) Representative AFM images illustrating the morphology of EVs in the preparations derived from KCL22 and K562 cell lines.

EVs were separated from the conditioned media of K562 and KCL22 cell lines (10 mL of starting material, from at least 2 × 10^8^ cells per line), using a commercial kit based on a silicon carbide resin matrix as described in the materials and methods section. The kit was chosen as a simple, time‐saving, and cheap EV concentration method [44]. These characteristics make this strategy one of the most suitable options for clinical translational applications [39]. According to the MISEV2023 guidelines [20], EVs were characterised from biochemical, biophysical, and morphological perspectives. Total protein concentration in the samples was determined with the BCA assay: K562 showed a mean protein concentration of 0.45 μg/μL, while KCL22 showed 0.31 μg/μL. The biochemical characterisation was performed using dot blot analysis as previously described [40]. As shown in Figure 2A, the specific EV markers ALIX and the membrane tetraspanin CD63 were detected in EVs isolated from both K562 and KCL22 cell lines. In contrast, TSG101 was undetectable, likely due to the low presence of this protein in both K562 and KCL22‐derived EVs. To assess the purity of the EV preparations and exclude contamination by cellular components, the presence of the Golgi marker GM130 was also evaluated. As expected, GM130 was strongly expressed in the cell homogenate used as a positive control but absent in EVs derived from KCL22. A faint signal for GM130 was observed in EVs from K562, though markedly lower than that in the homogenate (Figure 2A). These findings confirm the successful isolation of EVs and suggest minimal contamination by cellular debris only for K562 EVs.

Biophysical characterisation of EVs was performed using NTA, which enabled the evaluation of size distribution and particle concentration in samples from both cell lines. EVs from K562 displayed a concentration of 4.79 × 10^9^ ± 2.38 × 10^8^ particles/mL and a size distribution from 30 to 430 nm with a smaller mode size of 114.5 nm. EVs derived from KCL22 showed a mean concentration of 9.16 × 10^8^ ± 2.79 × 10^7^ particles/mL and a size distribution from 30 to 315 nm with a mode diameter of 143.6 nm (Figure 2B). NTA results highlighted that K562 and KCL22‐derived EVs have statistically significant diameters, with KCL22 releasing bigger particles than the K562 cell line (Student's t‐test; *p* = 0.000144). Particle concentration was not compared between the two cell lines due to the different number of cells used for EVs harvesting and the different cellular doubling time.

The presence of EVs was further confirmed by morphological analysis of the preparation using AFM. As shown in Figure 2C, AFM imaging revealed round‐shaped particles with diameters of hundreds of nm, consistent with those expected for EVs. These size measurements were also in agreement with the data obtained from NTA. In addition, AFM imaging revealed that both preparations do not present traces of residual matrix derived from the kit separation. The image background is uniformly black for both K562 and KCL22 derived samples, presenting a surface roughness close to 0 nm (Figure 2C). These data are in contrast with previous results obtained from other types of EV‐separation kits, based on polymers [45]. EV preparations obtained using polymers frequently exhibit adsorption of globular or spotted residues on the mica surface, which are indicative of co‐isolated polymeric contaminants or soluble protein impurities within the EV preparations. These findings suggest that the kit used in this study, based on silicon carbide resin, offers superior performance in terms of purity from co‐isolated residues.

### 3.2 K562 and KCL22‐Derived EVs Present Different Molecular Fingerprint With FTIR Spectroscopy

**FIGURE 3:**
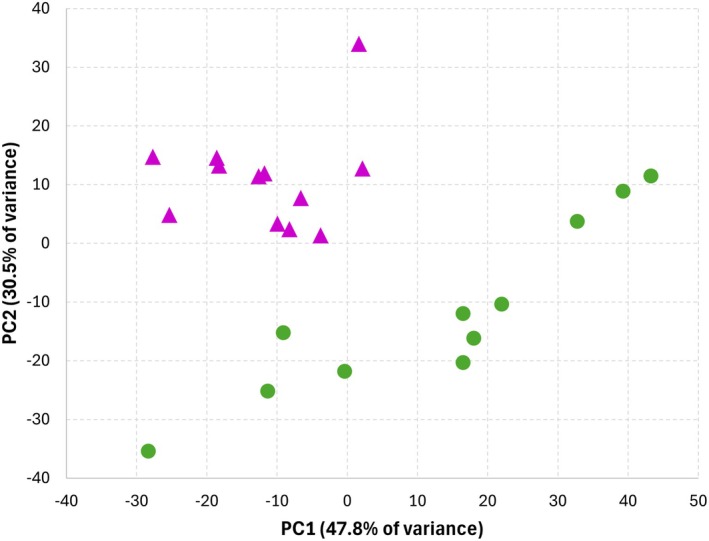
PCA analysis of EVs derived from K562 and KCL22 cell line visualised in Score plot.

To evaluate the molecular fingerprint of the different EVs, samples were analysed by FTIR as described in Material and methods Section 2.2. Principal Component Analysis (PCA), as an unsupervised technique, was performed to evaluate the overall spectral variability among EV samples and to assess whether the spectral fingerprint of EVs differs between the two cell lines. PCA reduces the dataset dimensionality of FTIR spectra by transforming them into principal components that capture the most relevant variance, enabling the identification of sample clustering based on intrinsic spectral characteristics. The analysis revealed a clear separation of K562 and KCLL2‐derived EVs into two distinct clusters (Figure 3), with PC1 and PC2 accounting for 47.8% and 30.5% of the total variance, respectively. This pattern suggests that EVs derived from the same cell line share common features while exhibiting systematic differences when compared to EVs derived from a different cell line. Such clustering may reflect underlying biological or biochemical variability relevant to the conditions being studied.

### 3.3 Cellular *Ki67* Transcript Expression Following TKIs Treatment

**FIGURE 4:**
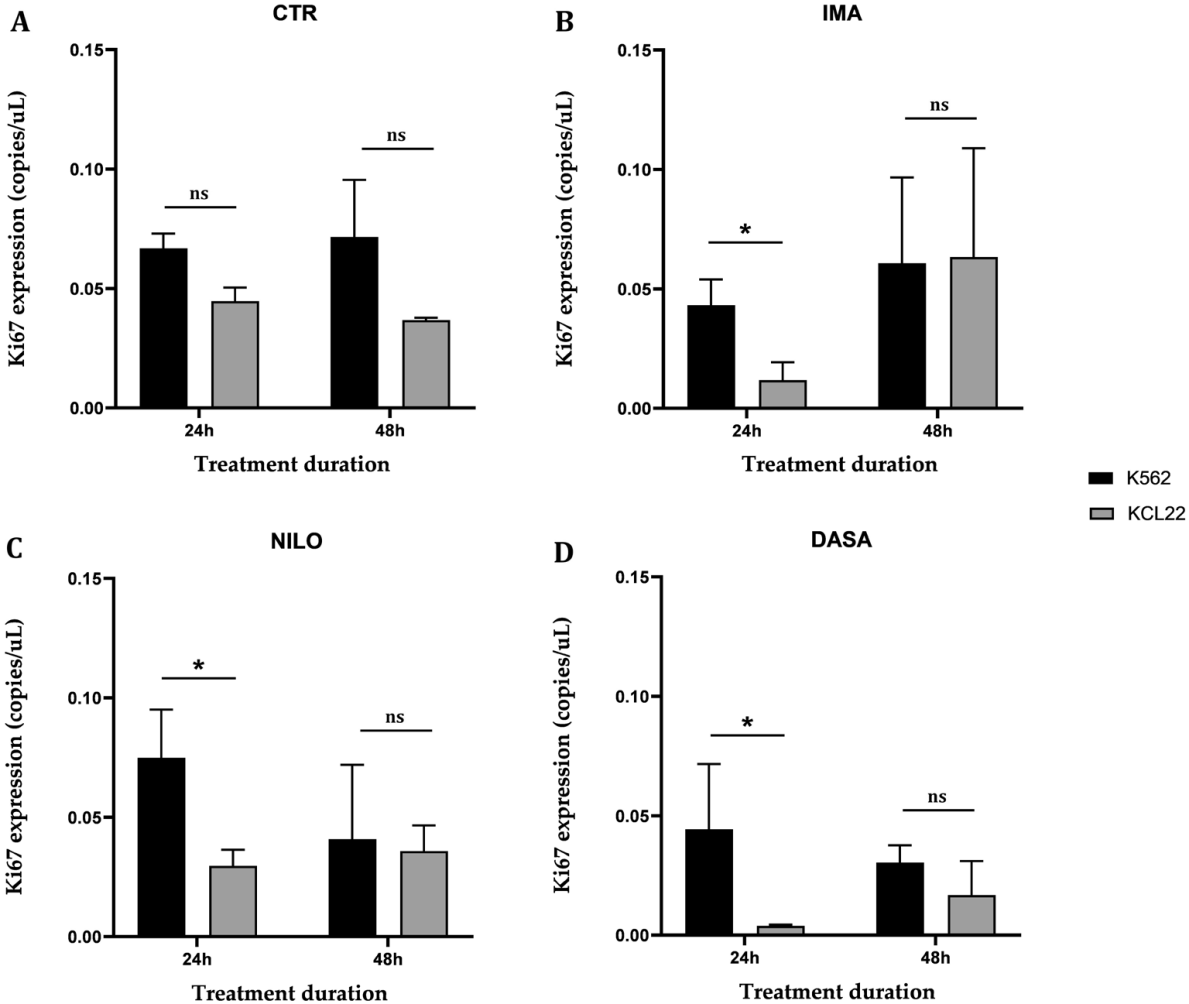
Expression of cellular Ki67 transcript (normalised to GAPDH) in K562 and KCL22 cell lines after 24 and 48 h of treatment, representing the comparison across individual treatment conditions (CTR, control; DASA: dasatinib; IMA, imatinib; NILO, nilotinib) (One‐way ANOVA with Sidak's multiple comparisons test. ns, not significant; *p < 0.05).

To account for differences in proliferative responses that might impact EVs biogenesis and release, the proliferative marker *Ki67* was evaluated and normalised to the housekeeper *GAPDH* to correct pre‐analytical variability during extraction and reverse transcription. The results of *Ki67* expression in untreated cells (CTR) show no differences between the two cell lines at both 24 h and 48 h. At 24 h, the expression level of *Ki67* in the KCL22 cell line is significantly lower when compared to K562 in response to treatment with imatinib (IMA, *p* = 0.0144), nilotinib (NILO, *p* = 0.0211), and dasatinib (DASA, *p* = 0.021). After 48 h, *Ki67* expression is similar between cell lines in all treatments, with no significant differences between them (Figure 4).

### 3.4 *BCR::ABL1* Transcript Expression in EVs Following TKIs Cellular Treatment

The expression of the disease hallmark *BCR::ABL1* in EVs was analysed using dPCR. Notably, the analysis did not include normalisation of expression levels to a reference gene. This approach was chosen given the absence of a universally accepted and conserved marker for EV normalisation, as no such marker has been identified to date, to the best of our knowledge. However, to account for differences in doubling times between the two cell lines, the absolute expression of the vesicular transcript was normalised to the total cell number for each treatment and time point.

**FIGURE 5:**
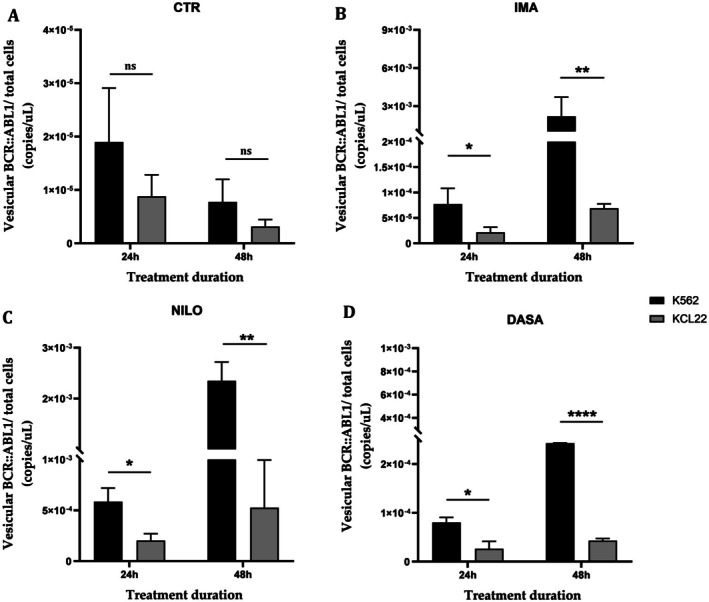
Expression of BCR::ABL1 transcript (normalised to the total cell number) in EVs preparation from K562 and KCL22 cell lines after 24 and 48 h of treatment, representing the comparison across individual treatment conditions (CTR, control; DASA, dasatinib; IMA, imatinib; NILO, nilotinib) (One‐way ANOVA with Sidak's multiple comparisons test. ns, not significant; *p < 0.05; **p < 0.01; ****p < 0.0001).

The levels of vesicular *BCR::ABL1* transcript are comparable between the two cell lines, K562 and KCL22, in the control groups (CTR) at both time points analysed (24 and 48 h) (Figure 5A). However, following imatinib, nilotinib and dasatinib treatments, K562 cells show a higher transport of vesicular *BCR::ABL1* transcript levels compared to KCL22 cells at both time points, with increased significance observed at 48 h (IMA, K562‐24h vs. KCL22‐24h, *p* = 0.0413; K562‐48h vs. KCL22‐48h, *p* = 0.0072; NILO, K562‐24h vs. KCL22‐24h, *p* = 0.0212; K562‐48h vs. KCL22‐48h, *p* = 0.0061; DASA, K562‐24h vs. KCL22‐24h, *p* = 0.0228; K562‐48h vs. KCL22‐48h, *p* < 0.0001) (Figure 5B–D).

### 3.5 EVs Quantification Following TKIs Treatment

**FIGURE 6:**
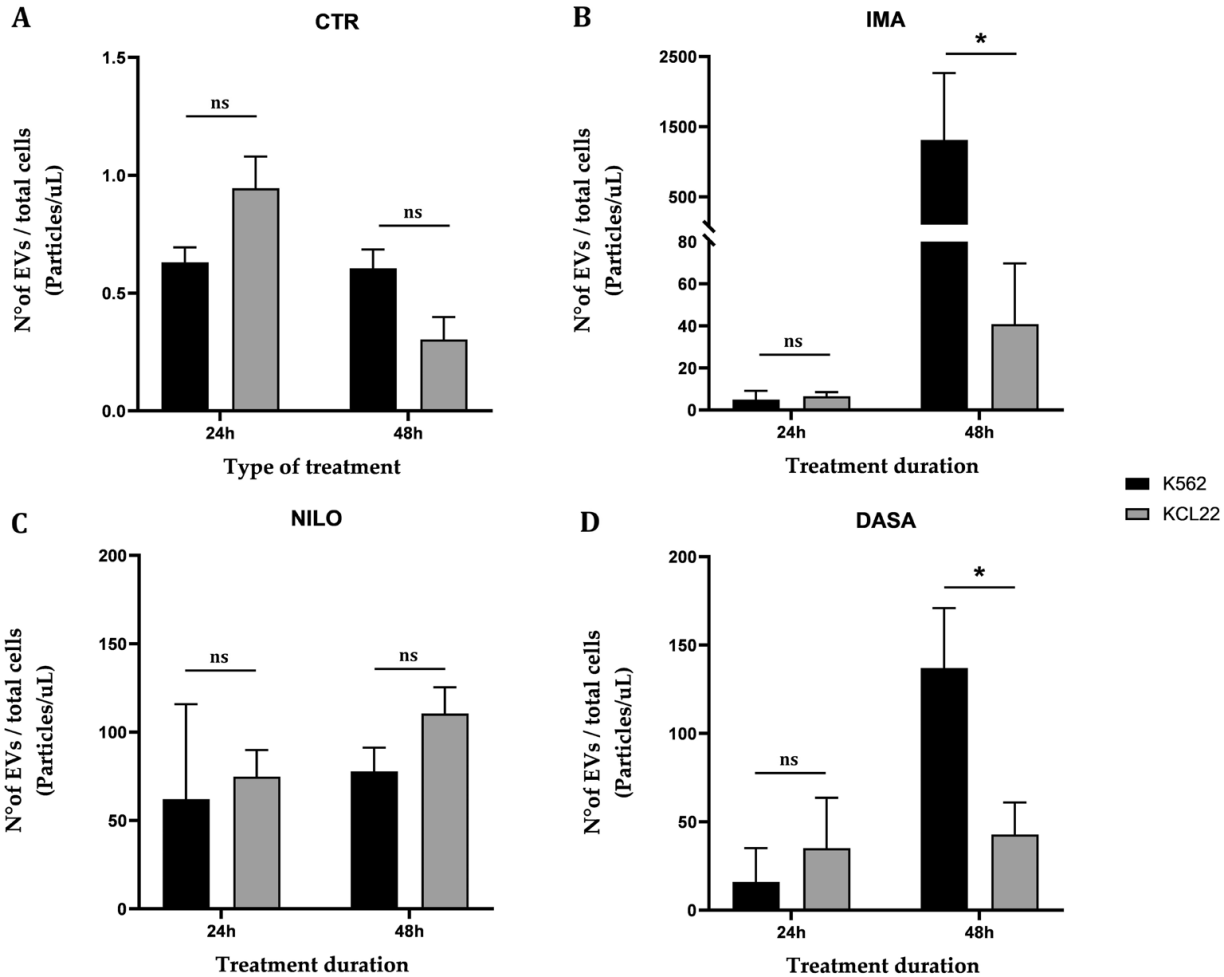
Quantification of the number of EVs (normalised to the total cell number) in K562 and KCL22 cell lines after 24 and 48 h of treatment, representing the comparison across individual treatment conditions (CTR, control; DASA, dasatinib; EVs, extracellular vesicles; IMA, imatinib; NILO, nilotinib) (One‐way ANOVA with Sidak's multiple comparisons test. ns, not significant; *p < 0.05).

EVs quantification was performed using NTA. Again, to account for differences in doubling times between the two cell lines, the absolute number of EVs was normalised to the total cell number for each treatment and time point. The results confirm that untreated cells (CTR) show no differences in EVs count between the two cell lines at both 24 and 48 h (Figure 6A). Similarly, treatment with nilotinib shows no differences at either time point (Figure 6C). However, significant differences arise between the two models after 48 h of treatment with imatinib (*p* = 0.0229) and dasatinib (*p* = 0.0246), with K562 cells showing a higher EVs count (Figure 6B,D).

## 4 Discussion

Chronic Myeloid Leukaemia (CML) is a myeloproliferative disorder originating from the haematopoietic stem cell compartment. Its etiopathogenesis is attributed to the balanced translocation t(9;22), involving the BCR gene (ch. 22) and the ABL1 gene (ch. 9), leading to the formation of the *BCR::ABL1* oncogene and its corresponding and constitutively active protein. Currently, targeted therapy with TKIs has transformed CML from a life‐threatening disease into a manageable chronic condition, allowing most patients to achieve nearly normal life expectancy. These treatments changed the prognosis of most CML patients who can achieve a nearly normal life expectancy [46, 47, 48]. However, challenges such as side effects, noncompliance, and resistance to TKIs persist in some patients, underscoring the need to address unmet clinical demands. Developing reliable preclinical in vitro models is critical for translating laboratory findings into clinical applications. Among the many available Ph + cell lines, these models provide valuable tools for studying different leukaemic states beyond chronic‐phase CML and are commonly regarded as biological replicates. As a result, it is standard practice in scientific experimentation to use a single in vitro model as the primary biological representation of a disease pathology. However, in a recent study, Cavalleri et al. highlighted significant differences among three frequently used CML models in research: K562, LAMA84, and KCL22 cell lines. This underscores the importance of selecting appropriate in vitro models for preclinical research, considering the cellular origin, genetic background, and phenotypic characteristics of each cell line when designing experiments, as they reflect the inter‐patient variability [11]. In accordance with these findings, we propose that further investigation into differences at the EV level across CML cell lines would be highly valuable, even in cases where the cell lines exhibit similarity, are derived from patients with comparable characteristics, and originate from the same biological compartment (e.g., K562 and KCL22). Leukaemic EVs in CML play a critical role in disease biology by modulating the immune response, promoting angiogenesis, facilitating metastasis, and transferring bioactive molecules, including the *BCR::ABL1* fusion gene [33], which may contribute to disease progression and therapy resistance. However, limited studies have explored the EV‐release profiles of drug‐sensitive CML cell lines under TKI treatment, and no comparisons have been made between EV release and cargo among different cell lines. As a result, the present study aimed to evaluate differences between K562 and KCL22 CML cell lines at the vesicular level following TKI treatment. EVs were separated from the two‐cell line conditioned medium, and the EV cargo was examined for the presence of *BCR::ABL1*. Additional analyses focused on the evaluation of the cellular proliferation marker *Ki67*. EV preparations from both cell lines were characterised according to MISEV guidelines for their size, concentration, morphology, and presence of typical EV markers and purity from cellular debris [19, 20]. Results showed differences in the protein composition of the two EV subpopulations, with KCL22‐EVs showing a higher signal intensity of the protein tetraspanin CD63 compared to K562‐EVs. In addition, the size of the EVs presented significant differences, with KCL22‐derived EVs having a higher diameter than K562‐derived EVs.

We previously demonstrated that FTIR spectroscopy combined with multivariate statistical analysis is a powerful tool to assess the presence of intrinsic molecular differences among EVs from different cell lines [25]. We applied this strategy to our samples, and data confirmed the results obtained with the biochemical characterisation via dot blot, highlighting the presence of underlying biological variability between K562 and KCL22‐derived EVs. The converging results obtained from different bio‐orthogonal techniques confirmed that although both cell lines originate from pleural effusions of CML patients, they cannot always be considered biologically equivalent.

This emphasises the importance of selecting CML cell lines together with the potential of advanced analytical tools to uncover biological variability. Upon TKIs treatment, the proliferation marker *Ki67* exhibited distinct temporal patterns between the two cell lines. *Ki67* is a well‐known marker of cell proliferation expressed during active phases of the cell cycle (G1, S, G2, and M) but absent in resting (G0) cells. Thus, when *Ki67* level drops, it reflects a shift toward reduced proliferation. While untreated cells showed no differences in *Ki67* expression, KCL22 cells demonstrated a significantly lower expression in *Ki67* levels at 24 h following imatinib, nilotinib, and dasatinib treatment when compared to the K562 cell line. This suggests that KCL22 cells may exhibit a more immediate sensitivity to TKIs in terms of cell cycle regulation compared to K562 cells. However, the absence of differences in *Ki67* expression between the two cell lines at 48 h implies a convergence in proliferative responses over time, possibly due to reduced cell cycle activity confirming treatment response as shown in clinical practice [49, 50]. Likewise, at the EV‐level, the expression of the *BCR::ABL1* transcript revealed key differences between the two cell lines under TKI treatment. While control conditions demonstrated comparable vesicular *BCR::ABL1* levels, treatment with imatinib, nilotinib, and dasatinib resulted in a higher transport of EV‐*BCR::ABL1* transcript in K562 cells compared to KCL22 cells. This effect was particularly pronounced at 48 h. The higher levels of EV‐*BCR::ABL1* in K562 cells may indicate an altered vesicle‐mediated intercellular communication in response to TKIs, potentially reflecting cell line‐specific mechanisms of disease progression, as observed in other pathological contexts [51, 52]. Moreover, KCL22 cells appeared more sensitive to TKI treatment, as demonstrated by a greater reduction in cellular proliferation based on *Ki67* analysis. Since EVs biogenesis pathways, such as endosomal sorting and plasma membrane budding, are regulated by intracellular signalling pathways, their activity may be downregulated when cell proliferation decreases. Thus, it is plausible that their downregulation in response to reduced proliferation contributes to the diminished vesicular transport of *BCR::ABL1* observed in KCL22 cells. In terms of EVs release, the NTA‐based analysis showed no differences in untreated cells, supporting the baseline similarity in EVs production between the two cell lines. However, significant differences emerged following imatinib and dasatinib treatment at 48 h, with K562 cells exhibiting higher EVs counts. This finding may suggest that K562 cells respond to certain TKIs by increasing vesicle release, potentially as a mechanism to expel therapeutic agents, modulate the tumour microenvironment, or facilitate intercellular signalling. Variation in extracellular EVs release was observed in many contexts following drug treatment [53, 54, 55, 56]. The lack of differences in EVs counts under nilotinib treatment may indicate a distinct mode of action or vesicular dynamics specific to this TKI.

Taking together, these results demonstrate that K562 and KCL22 cell lines exhibit distinct EVs and proliferative responses to TKIs treatment. The observed differences underscore the importance of considering cell line‐specific characteristics when interpreting preclinical findings and designing therapeutic strategies. In recent years, extracellular vesicles (EVs) have been increasingly recognised as valuable biomarkers and potential therapeutic tools. Therefore, it is crucial to acknowledge that the vesicular profiles of different cell lines may not accurately reflect the clinical setting, potentially limiting the translational relevance of certain in vitro findings. In conclusion, in line with our previous results, our study emphasises the critical need to choose appropriate in vitro models for preclinical research, with careful consideration of each cell line's cellular origin, genetic background, and phenotypic traits, as these factors mirror inter‐patient variability. While the results of this study are not intended for direct clinical application, they offer valuable insights that can inform future preclinical investigations.

## Author Contributions

**Silvia Mutti:** conceptualization, methodology, validation, formal analysis, investigation, data curation, visualization, writing – original draft, writing – review and editing, software. **Alessia Cavalleri:** conceptualization, methodology, software, data curation, investigation, validation, formal analysis, visualization, writing – original draft, writing – review and editing. **Stefania Federici:** investigation, formal analysis. **Valentina Mangolini:** resources, investigation. **Lucia Paolini:** resources, investigation, writing – review and editing. **Cristian Bonvicini:** resources, investigation. **Rosalba Monica Ferraro:** investigation. **Elena Laura Mazzoldi:** resources, investigation. **Luca Garuffo:** software, formal analysis. **Besjana Xhahysa:** formal analysis, software. **Alessandro Leoni:** software, formal analysis. **Federica Trenta:** software, formal analysis. **Federica Re:** software, formal analysis. **Silvia Clara Giliani:** resources. **Daniele Avenoso:** resources. **Mirko Farina:** resources. **Michele Malagola:** resources. **Domenico Russo:** resources, funding acquisition. **Simona Bernardi:** conceptualization, writing – review and editing, project administration, visualization, funding acquisition, supervision.

## Conflicts of Interest

The authors declare no conflicts of interest.

## Supporting information


**Data S1:** jcmm70901‐sup‐0001‐Supinfo.docx.

## Data Availability

Data will be made available on request.
