# Supplementary material for: Assessment of Chronic Myeloid Leukaemia In Vitro Models Variability: Insights Into Extracellular Vesicles
Source: J Cell Mol Med. 2025 Oct 22;29(20):e70901. doi: 10.1111/jcmm.70901 (PMC12541237; doi:10.1111/jcmm.70901)
Supplement: Supplementary file 1 — Data S1: jcmm70901‐sup‐0001‐Supinfo.docx. [file JCMM-29-e70901-s001.docx]

**Assessment of Chronic Myeloid Leukemia in-vitro models variability: insights into extracellular vesicles**

Silvia Mutti ^a, b,*,^ ^†^, Alessia Cavalleri ^a, b,*^, Stefania Federici ^c^, Valentina Mangolini ^d, e^, Lucia Paolini ^f, g^, Cristian Bonvicini ^h^, Rosalba Monica Ferraro ^i^, Elena Laura Mazzoldi ^i^, Luca Garuffo ^a, b^, Besjana Xhahysa ^a, b^, Alessandro Leoni ^a, b^, Federica Trenta ^a, b^, Federica Re ^a, b^, Silvia Clara Giliani ^i^, Daniele Avenoso ^a^, Mirko Farina ^a^, Michele Malagola ^a^, Domenico Russo ^a^, Simona Bernardi ^a, b, j^.

* The first two authors equally contributed to the article

†Corresponding author:   Silvia Mutti, University of Brescia, silvia.mutti@unibs.it, TEL: 0039 0303998464

1. Department of Clinical and Experimental Sciences, University of Brescia, Unit of Blood Diseases and Bone Marrow Transplant, ASST Spedali Civili, 25123, Brescia, Italy.
2. Centro di Ricerca Emato-Oncologica AIL (CREA), ASST Spedali Civili di Brescia, 25123, Brescia, Italy.
3. Department of Mechanical and Industrial Engineering, University of Brescia, Brescia, Italy; National Interuniversity Consortium of Materials Science and Technology (INSTM), Florence, Italy.
4. Department of Molecular and Translational Medicine (DMMT), Università di Brescia, Brescia, Italy.
5. IRCCS Fondazione Don Carlo Gnocchi ONLUS, Milan, Italy.
6. Department of Medical and Surgical Specialties, Radiological Sciences and Public Health (DSMC), Università di Brescia, Brescia, Italy.
7. Center for Colloid and Surface Science, (CSGI), Sesto Fiorentino (FI), Italy.
8. Molecular Markers Laboratory, IRCCS Istituto Centro San Giovanni di Dio Fatebenefratelli, Brescia, Italy.
9. Department of Molecular and Translational medicine, University of Brescia, “Angelo Nocivelli” Institute for Molecular Medicine, ASST Spedali Civili, Brescia, Italy.
10. National Center for Gene Therapy and Drugs based on RNA Technology (CN3), Padua, Italy.

**Supplementary materials**

**Materials and methods**

**Characterization and quantification of K562 and KCL22-derived EVs in basal condition**

For dot blot analyses, 6 or 3 ug of EVs were spotted on a nitrocellulose membrane and allowed to dry at RT for 1 h. Membranes were then blocked with 5% (w/v) fat-free dried milk in Tris-buffered saline with 0.1% (v/v) Tween-20 (TBST) for 1 h at 37°C, followed by the incubation overnight at 4°C with anti-CD63 (Millipore, clone RFAC4, CBL553), anti-ALIX (Santa Cruz Biotechnology, clone G-10, sc-166952), anti-TSG101 (Santa Cruz Biotechnology, clone C‑2, sc‑7964) and anti-GM130 (BD Transduction, clone 35/130, 610822) antibodies, diluted 1:500 in TBS 1% fat-free dried milk. After three washes with TBST, membranes were incubated with HRP-conjugated secondary antibodies diluted in TBST 1% fat-free dried milk for 1 h at RT. Again, membranes were washed three times with TBST, then, blots were detected on a G:Box Chemi XT Imaging system (Syngene).

**dPCR analysis of vesicular *BCR::ABL1* and cellular *Ki67* and *GAPDH* after TKIs treatment**

Five μl of EV samples were used for dPCR by mixing 1.8 μL of Absolute Q™ DNA dPCR Master Mix (5X), 0.45 μL of 20X TaqMan-MGB-FAM probe assay, 5 μL of cDNA, and 1.75 μL of nuclease-free water (Qiagen). Cellular samples were prepared by combining 1.8 μL of Absolute Q™ DNA dPCR Master Mix (5X), 0.45 μL of each 20X TaqMan-MGB VIC and CY5 probe assays, 5 μL of cDNA, and 1.3 μL of nuclease-free water to achieve a final reaction volume of 9 μL. Both reaction mixes were loaded into Absolute Q MAP16 plates with 15 μL of Absolute Q™ Isolation Buffer. Thermocycling conditions included an initial denaturation at 95°C for 8 minutes, followed by 43 cycles of 90°C for 15 seconds and 60°C for 1 minute, and a final extension step at 60°C for 2 minutes.
